# Do estimates of numerosity really adhere to Weber’s law? A reexamination of two case studies

**DOI:** 10.3758/s13423-020-01801-z

**Published:** 2020-09-18

**Authors:** Alberto Testolin, James L. McClelland

**Affiliations:** 1 Department of General Psychology, University of Padova, Via Venezia 12, 35131 Padova, Italy; 2 Department of Information Engineering, University of Padova, Via Venezia 12, 35131 Padova, Italy; 3 Department of Psychology, Stanford University, Stanford, CA 94305 USA; 4 Center for Mind, Brain, Computation and Technology, Stanford University, Stanford, CA USA

**Keywords:** : Numerosity estimation, Approximate number system, Weber’s law, Coefficient of variation, Scalar variability

## Abstract

Both humans and nonhuman animals can exhibit sensitivity to the approximate number of items in a visual array or events in a sequence, and across various paradigms, uncertainty in numerosity judgments increases with the number estimated or produced. The pattern of increase is usually described as exhibiting approximate adherence to Weber’s law, such that uncertainty increases proportionally to the mean estimate, resulting in a constant coefficient of variation. Such a pattern has been proposed to be a *signature characteristic* of an innate “number sense.” We reexamine published behavioral data from two studies that have been cited as prototypical evidence of adherence to Weber’s law and observe that in both cases variability increases less than this account would predict, as indicated by a decreasing coefficient of variation with an increase in number. We also consider evidence from numerosity discrimination studies that show deviations from the constant coefficient of variation pattern. Though behavioral data can sometimes exhibit approximate adherence to Weber’s law, our findings suggest that such adherence is not a fixed characteristic of the mechanisms whereby humans and animals estimate numerosity. We suggest instead that the observed pattern of increase in variability with number depends on the circumstances of the task and stimuli, and reflects an adaptive ensemble of mechanisms composed to optimize performance under these circumstances.

A key finding in numerical cognition is that humans and nonhuman animals are able to make approximate judgments of numerosity, an ability often called “the number sense” (Dehaene, 2011). This phenomenon has been mostly investigated using discrimination or comparison tasks (Price, Palmer, Battista, & Ansari, 2012): If the number of items in two visual displays is very different, humans and animals can determine which display has more items with high accuracy, but accuracy decreases as difference in number decreases. Holding difference constant, accuracy also decreases as the number of items in the two displays increases. Discriminability of two numerosities can therefore often be characterized, at least approximately, as a function of their *ratio* (Dehaene, 2003; Gallistel & Gelman, 2000) or, equivalently, by the idea that uncertainty (as reflected in the standard deviation of the noise or variability in the representation of the number) is an approximately constant fraction of its magnitude, a relationship often expressed as *Weber’s law* (Halberda, 2011). Similar findings have been observed in adaptation (Burr & Ross, 2008) and match-to-sample (Ditz & Nieder, 2016; Merten & Nieder, 2009) studies.

A different approach is taken in estimation paradigms, where subjects are required to explicitly estimate the numerosity of items in a display (Izard & Dehaene, 2008; Revkin, Piazza, Izard, Cohen, & Dehaene, 2008) or in production studies, were subjects are required to produce a specified number of responses without counting (Platt & Johnson, 1971; Sella, Berteletti, Lucangeli, & Zorzi, 2015). With training or calibration, mean estimates can increase approximately linearly with the value being estimated (Izard & Dehaene, 2008), and estimation variability grows with number of items, often approximately proportionally to the mean (Whalen, Gallistel, & Gelman, 1999). In these paradigms it is common to measure adherence to Weber’s law by computing the coefficient of variation (CV), which corresponds to the standard deviation of the response values divided by the mean, and which should be constant if responses follow Weber’s law.

The finding that uncertainty in the representation of the number of items increases in proportion to the number itself has been taken as a *signature characteristic* of the so-called approximate number system (Dehaene, 2003; Gallistel & Gelman, 2000), and has been used to support claims of ontogenetic and phylogenetic continuity of numerosity perception (Feigenson, Dehaene, & Spelke, 2004). According to this view, the ability to estimate number enhances individual fitness, and natural selection led to its early emergence and preservation across much if not all of the animal kingdom (Butterworth, 1999; Cantlon & Brannon, 2007; Ferrigno & Cantlon, 2017; Nieder, 2005; Wynn, 1998).

In this article, we revisit some of the evidence relevant to whether adherence to Weber’s law—or equivalently a constant CV—is a robust and universal characteristic of numerosity judgments, ultimately considering estimation, production, and discrimination tasks. As we will see, empirical findings do not always follow this pattern. We also consider the recent proposal that the observed departure from Weber’s law in numerosity discrimination tasks could be attributed to the existence of two separate mechanisms, one based on pure numerosity, which follows Weber’s law and operates over relatively sparse visual displays, and another based on texture information, which does not follow Weber’s law and prevails for denser stimuli (Anobile, Cicchini, & Burr, 2014; Pomè, Anobile, Cicchini, & Burr, 2019).

We focus on two studies that have been often cited as supporting adherence to Weber’s law in the case of stimuli with relatively sparse numerosities. First, we consider the numerosity estimation results reported by Revkin et al. (2008). In this study, participants estimated the numerosity of displays containing 1 to 8 dots in one session and estimated the numerosity of displays containing 10 to 80 dots in a different session. The authors concluded that the data from the larger range were consistent with Weber’s law, attributing the apparent deviation from this pattern (see Fig. 1) to edge effects, which reduce variability for judgments at both ends of the range. However, the decrease seems to start already from *n* = 40, suggesting that other factors might be contributing to the observed trend. In conjunction with this, we also consider an experiment by Newman (1974) investigating numerosity discrimination over the same range, using a method that is free from the range restriction effects influencing the study by Revkin et al. (2008). Second, we consider the animal data originally published by Platt and Johnson (1971), which has been analyzed and presented by Gallistel and Gelman (2000) as a textbook case for perfect adherence to Weber’s law. This experiment has also been considered important because a constant CV pattern has been sometimes observed in human studies (Whalen et al., 1999); a similar pattern in rodents therefore serves to support the view that numerosity production taps a primitive, phylogenetically preserved mechanism with the same signature characteristic as that purported to hold for perception of the numerosity of dots. In all three cases, we carefully reexamine the pattern of behavioral responses, to test the hypothesis that the distributions of numerosity estimates might not have a constant CV.

**Fig. 1:**
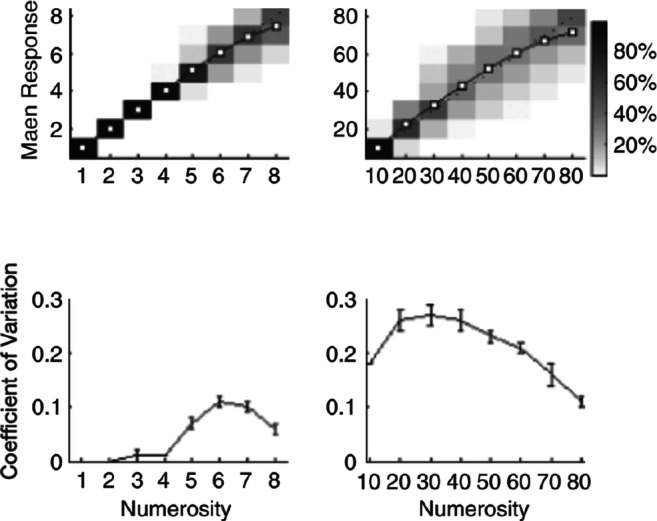
Human numerosity estimation data. Upper panels show mean responses, where the gray shading indicates response frequency in relation to the total number of responses given for each numerosity (see scale with percentages on the right). Lower panels show the estimated coefficient of variation. Reprinted from Revkin et al. (2008)

## Deviations from conformity to the “signature characteristic”

### Human numerosity estimation

As noted above, Revkin et al. (2008) tested adults with numerosities in the ranges 1 to 8 and 10 to 80. Participants were restricted to using the responses 1 to 8 in the first condition and the decadal responses 10 to 80 in the second condition. In both tasks, there were calibration trials not used for analysis prior to the trials included in the analysis, and throughout the experiment, the correct numerosity was provided as feedback if the response was incorrect. Numerosity estimation responses were fast and virtually error-free for *n =* 1 to 4, while estimates were slower and much more errorful for larger numerosities. The means and distributions of responses for each numerosity are displayed in the upper panels of Fig. 1, while the corresponding CVs are shown in the lower panels. We focus on the CV over the 10–80 range, which, as mentioned above, exhibited an inverted U-shape trend that the authors attributed to range restriction effects. While we agree that range restriction is necessarily in play (responses could not go outside the 10–80 range), modeling the effect of range restriction seems necessary to determine whether the underlying CV is in fact constant over the indicated range.

#### Modeling the behavioral data

We estimated the mean estimation response for each numerosity, as well as the CV and standard error of the CV from Fig. 3 of Revkin et al. (2008)*,* using computer graphics software.^1^ To determine whether the observed variation in the CV with numerosity for *n* in the range from 10 to 80 could be attributed to range restriction effects, we implemented computer simulations that allowed us to compare different models describing the relationship between the numerosity estimate and the CV. The crucial question was whether the overt pattern of CV estimates could be based on an underlying CV that is a constant function of the underlying mean estimate, or equivalently, whether the standard deviation of underlying estimates, *sd*(*n*), is a scalar function of the mean underlying estimate, *mu*(*n*), that is *sd*(*n*) = *s*_*sd*_ × *mu*(*n*). We call models with this feature “scalar variability” models. Alternatively, we consider the possibility that the standard deviation of underlying estimates might better be explained by assuming that they increased according to a power function of the mean of the underlying estimate.

To explore this, we examined a set of four nested models, in which numerosity estimates were simulated by assuming participants derived underlying estimates of numerosity on each trial that were drawn from a Gaussian distribution whose mean depended on the actual presented numerosity *n* and whose standard deviation depended on the mean for the given value of *n.* Our approach allows the mean to be fitted by a fairly general monotonic increasing function:

1 $$ mu(n)={s}_{mu}\times {n}^{p_{mu}}+ off. $$

With *sd* following the functional form:

2 $$ sd(n)={s}_{sd}\times mu{(n)}^{p_{sd}}, $$

where *s*_*mu*_ and *s*_*sd*_ represent scaling factors, *p*_*mu*_ and *p*_*sd*_ represent power exponents, and *off* is an additive offset. This general formulation allows us to explore the possibility that both the mean and the standard deviation could follow scalar or power trends by imposing specific constraints on the fitting procedure:

- *p*_*mu*_ = *p*_*sd*_ = 1 results in a “linear mean, scalar variability” model;
- *p*_*mu*_ = 1 results in a “linear mean, power variability” model;
- *p*_*sd*_ = 1 results in a “power mean, scalar variability” model;
- with no constraints, we obtain a “power mean, power variability” model.

We also considered two additional models that tested the hypothesis that for displays above a certain density, response variability decreases as a square root of numerosity, reflecting a shift to using texture information to judge numerosity, with the standard deviation of texture estimation assumed to follow a square root law (Anobile et al., 2014; Pomè et al., 2019). It should be noted that these models require that the density of the displays increases with numerosity in the experiment by Revkin et al. (2008). However, information presented in the article and personal correspondence with authors of the paper failed to resolve the question of whether in fact density increased with numerosity in all of the displays used in the study: density may have been approximately constant in one-half of the displays, but not the other half (see p. 609, col 2, lines 200–22, in Revkin et al., 2008). Despite this ambiguity, it seemed worthwhile to consider whether these models could help explain the observed pattern of the data. In this case, the *sd* equation includes an additional parameter *k*_*sd*_:

3 $$ sd(n)=\min \left({s}_{sd}\times mu{(n)}^{p_{sd}},{k}_{sd}\times \sqrt{n}\right). $$

In principle, also for the density regimen, the *sd* could be parameterized on the actual mean *mu* rather than on the true numerosity *n*. We explored this alternative, which produced qualitatively similar results to those obtained using the true numerosity; here, we report the original approach to maintain full compatibility with the model proposed by Burr and colleagues (Anobile et al., 2014). As we shall see, fits to the mean estimates were excellent under the power mean model variants of the first four models, so in this case we did not consider the linear mean model variants. The two variants considered were thus sparse versus dense regimen, scalar variability and sparse versus dense regimen, power variability.

For a given model variant and set of parameter values, the mean and CV of the observed responses relied on the estimated mean and standard deviation of the underlying Gaussian distribution for each presented numerosity, subject to restrictions imposed by the eight response categories used in the experiment (10, 20, 30, 40, 50, 60, 70, 80). The underlying assumption was that participants in Revkin et al. (2008) had placed response boundaries along their subjective numerosity continuum halfway between the available category labels. The probability of a response falling into a given response category was determined using the cumulative density function of the Gaussian for the presented numerosity, using the midpoints between the eight response categories as boundaries (15, 25, 35, 45, 55, 65, 75).^2^

The best fitting values for the free parameters were found by minimizing the sum squared error (SSE) between the empirical data and the model predictions both for the mean estimates and the CV. To compare the goodness of fit among the models to the observed data, we also report log likelihood (LL) values, along with a distribution of log likelihood values based on 1,000 simulated data sets generated from each model using its best fitting parameter values. This allowed us to calculate an index *p* that indicates the probability that the log likelihood of the empirical data could have been observed if the data had been generated from the model using these parameters (for details, see the Appendix).

#### Results and discussion

**Fig. 2:**
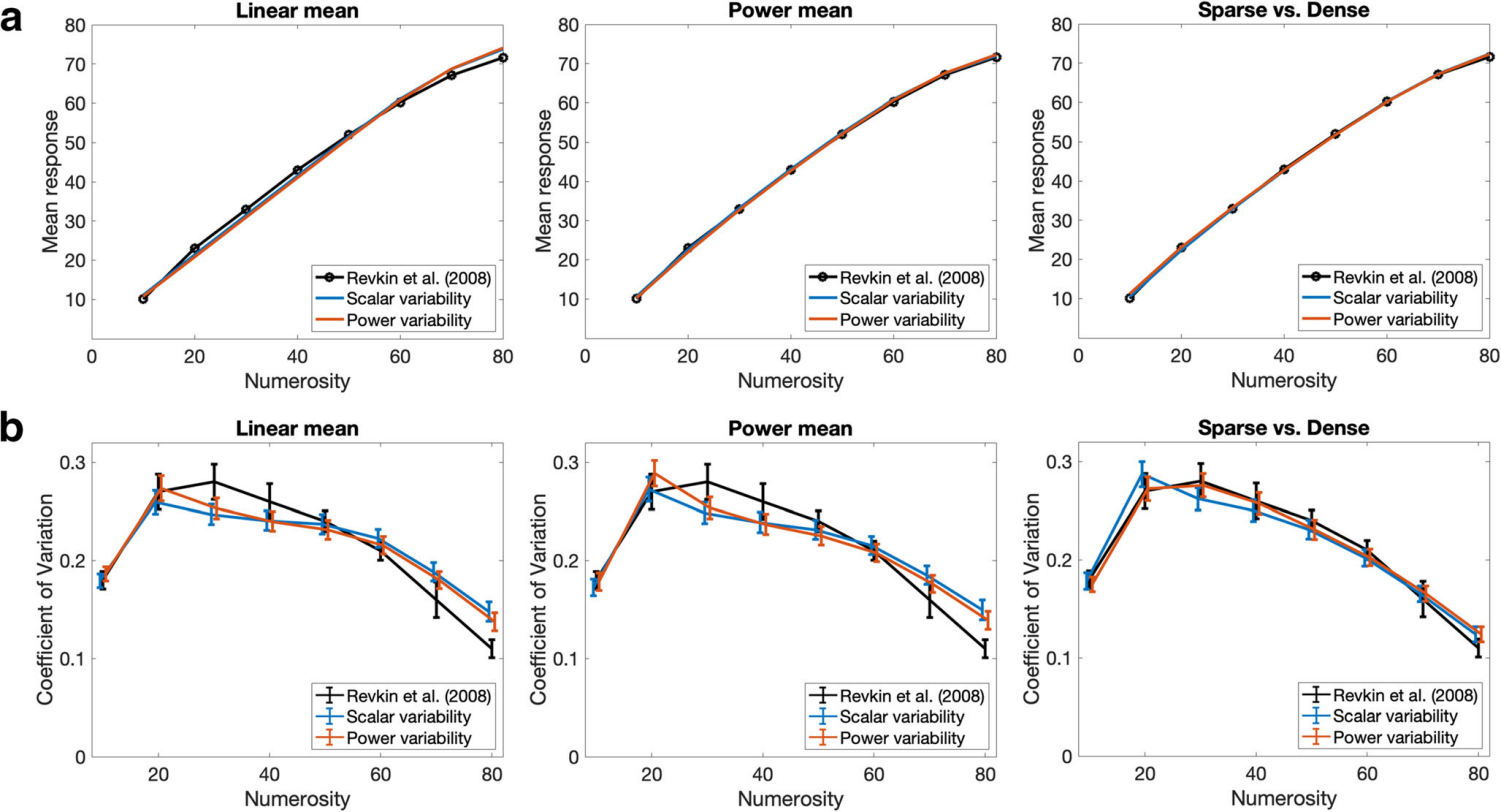
**a** Predicted mean responses for each numerosity category considered in the Revkin et al. (2008) study. The original empirical curve is shown in black. **b** Predicted coefficient of variation (CV) trend. The empirical curve is shown in black (the standard error for *n* = 10 is not visible in the original figure, so we considered it being equal to that of *n* = 80 because the authors reported that variability was very small for these extreme values). In all panels, the blue and red curves correspond, respectively, to simulations with a response model featuring scalar variability (constant CV) or power variability. (Color figure online)

The resulting parameters and goodness of fit statistics for all models are reported in Table 1. The trend of mean estimates is very well captured by power mean models, as indicated by the smaller SSE for *mu* and by the curves shown in top panels of Fig. 2. The inverted U-shape trend of the CV is approximately captured by all models; however, the fit is acceptable only for the models including sparse and dense regimens, which indeed obtain smaller SSE (and higher LL) for the CV. In fact, only these models could produce samples with LL values reliably falling in the range of the empirical data, as indicated by values of *p* greater than zero.

**Table 1:** Parameters and goodness of fit statistics for the models describing the data from Revkin et al. (2008)

Model type	*s*_*mu*_	*p*_*mu*_	*off*	*s*_*sd*_	*p*_*sd*_	*k*_*sd*_	SSE *mu*	SSE CV	LL	*p*
Linear mean, scalar variability	1.00	1	0.62	0.24	1	–	16.10	0.0041	12.46	0.00
Linear mean, power variability	1.01	1	0.26	0.32	0.91	–	23.51	0.0026	18.39	0.00
Power mean, scalar variability	1.66	0.89	−2.58	0.24	1	–	1.56	0.0039	12.37	0.00
Power mean, power variability	1.69	0.89	−3.13	0.33	0.91	–	2.27	0.0030	17.27	0.00
Sparse *vs*. dense regimen, scalar variability	1.73	0.88	−2.97	0.26	1	1.76	1.65	0.0011	24.05	0.25
Sparse *vs*. dense regimen, power variability	1.56	0.90	−1.51	0.12	1.25	1.76	2.15	0.0005	24.96	0.64

*Note.* The SSE is reported separately for the mean and for the CV (the lower the better). The last two columns report log likelihoods (the higher the better) and the related proportion *p* of simulations in the range of empirical data, estimated using the simulation procedure described in the Appendix

We now consider what these results have to tell us about the variability in participants’ underlying estimates of numerosity. In one way, the superior fit of the sparse versus dense regimen models indicates that numerosity estimates may not rely on a variable reflecting scalar variability throughout the range from 10 to 80. Instead, numerosity estimates in part of this range might actually be based on an estimate of density, which is subject to a different pattern of variability. Such a situation is far different from the one envisioned by, for example, Izard and Dehaene (2008), in which estimates are always considered to be based on numerosity per se. However, there remains the possibility, defended by Anobile et al. (2014), that there is still a pure numerosity system exhibiting adherence to Weber’s law, which determines responses in the range up to the point where reliance on density results in less variability of estimates than reliance on numerosity. Our simulations do not rule out this possibility. However, it should be noted that we obtained a better fit to the CV data in the sparse *vs.* dense regimen model under the power variability variant than under the scalar variability variant (SSE = .011 for scalar variability, .005 for power variability; also compare blue and red curves to the black curve in Fig. 2, bottom-right panel), suggesting that even in the sparse regime scalar variability might not adequately characterize the underlying variability in participants numerosity estimates. A further caveat is that density may have increased with numerosity only for half of the stimuli, making the applicability of the sparse versus dense regimen to the data less than fully clear. We therefore considered it useful to examine another data set free from this ambiguity, and also free from the range restriction effects influencing the estimation judgments in Revkin et al. (2008).

#### Relevant data from a discrimination paradigm

The additional relevant evidence is provided by an earlier experiment by Newman (1974), where participants carried out a numerosity discrimination task in which dots of uniform size were placed at random within a rectangular space of fixed size independent of *n* (subject only to the constraint that the dots not touch or overlap each other or the rectangle bounding the space). In this case, density increases with *n,* allowing an assessment of the models in a regime where the assumptions of the sparse versus dense regimen account apply. We thus considered the CV values reported by Newman and implemented a maximum likelihood estimation approach to fit the different models described above to the empirical data. The log likelihood for each model was defined as for the data from Revkin and colleagues (see the Appendix), considering as the scaling factor *s* the values of the standard deviation of CV estimates reported by Newman, divided by the squared root of the number of participants. Because in this case there are no mean numerosity estimates provided, we only consider models in which CV is dependent on the true numerosity *n*. This leaves four models: The simple scalar variability model, the simple power variability model, and the two models featuring the sparse versus dense regimens.

**Fig. 3:**
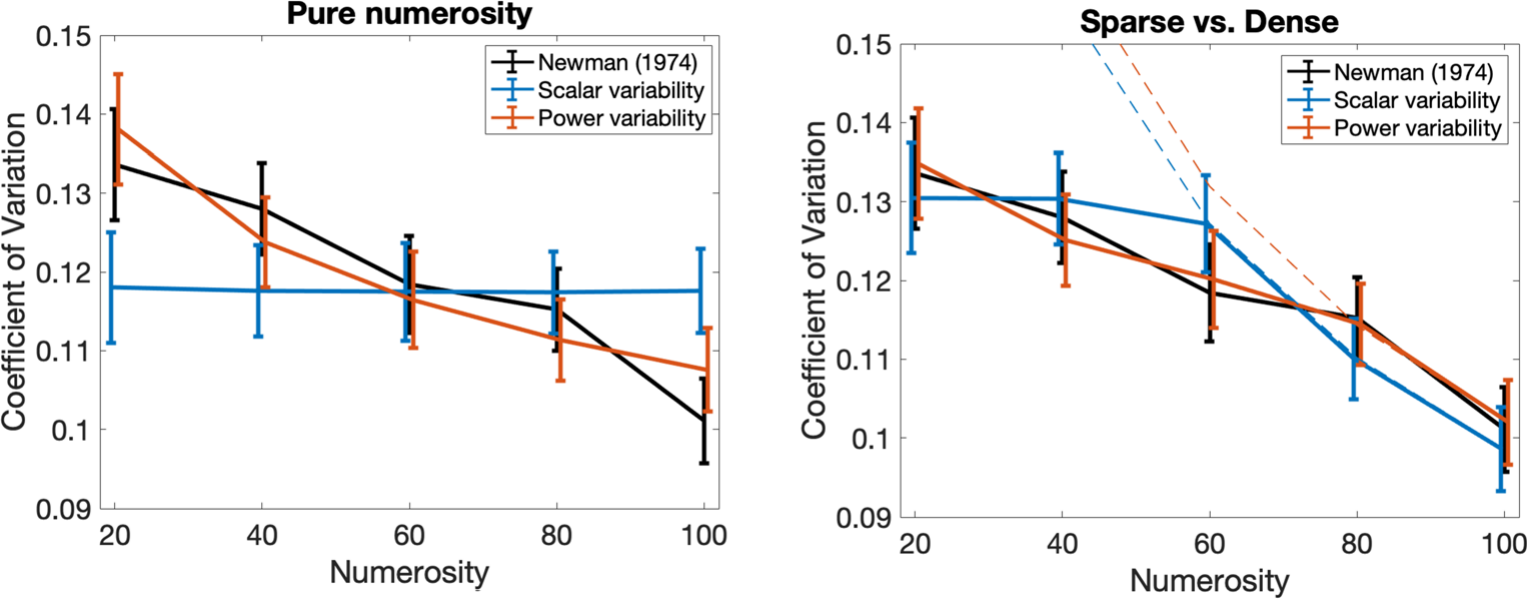
Predicted coefficient of variation (CV) trend of the various models for the data reported by Newman (1974). The empirical curve is shown in black. The error bars represent the standard error of the estimate of the CV, determined by dividing the standard deviation of each CV as reported by Newman (1974) by 8, the square root of the number of participants. In the right panel, dashed lines represent the estimated CV trend for the density regimen.

As shown in Fig. 3, the data reported by Newman (1974) is clearly inconsistent with the simple scalar variability model, which predicts a flat CV, invariant with *n.* Indeed, Newman’s own analysis showed a highly significant linear trend, ruling out the simple scalar variability model. We find that the data are best fit by the two models featuring power variability. The power variability model with sparse versus dense regimen achieved a near-perfect fit to the actual data points (see Table 2). The scalar variability model with the sparse versus dense regimen fits slightly less well than the two power models, but cannot be ruled out by our modeling approach. The pattern from this analysis is similar to the pattern observed in our simulations of Revkin et al. (2008): The simple scalar variability model can be ruled out; the power variability model with sparse versus dense regimen produces the closest fit; and models with either power variability, or scalar variability with the sparse versus dense regimen fit slightly less well. It is worth noting that for the study of Revkin and colleagues, the fit for the power variability model with sparse versus dense regimen resulted in *p*_*sd*_ > 1, allowing the estimated CV to be smaller than it would otherwise be for small numbers. Although this makes sense in terms of the fit to the data, further research will be required to better characterize the factors underlying this trend: Details of visual stimuli likely play a critical role, since they also contribute in defining the transition point where density information becomes dominant.

**Table 2:** Parameters and goodness of fit statistics for the models describing the data from Newman (1974)

Model type	*s*_*sd*_	*p*_*sd*_	*k*_*sd*_	SSE CV	LL	*p*
Scalar variability	0.12	1	–	6.44e-4	11.97	0.01
Power variability	0.22	0.85	–	0.97e-4	19.60	0.71
Sparse versus dense regimen, scalar variability	0.13	1	0.99	1.26e-4	19.31	0.61
Sparse versus dense regimen, power variability	0.18	0.90	1.02	0.01e-4	20.91	0.99

### Animal numerosity production

**Fig. 4:**
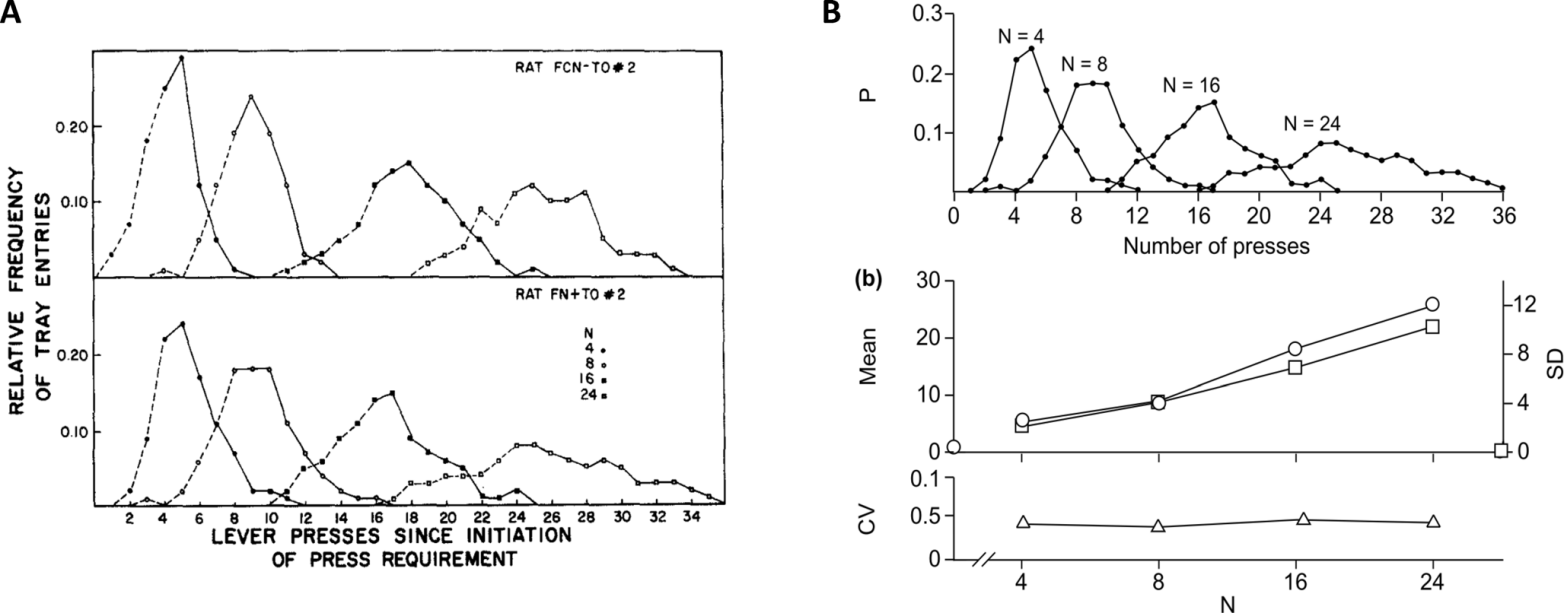
**a** Numerosity estimation data for two rats from the production study considered in our analysis. Reprinted from Platt and Johnson (1971). **b** Data for the second rat (top panel) along with mean, standard deviation (*SD*), and coefficient of variation (CV) as estimated by Gallistel and Gelman (2000). Note that values in the *x*-axis in the bottom panel are not linearly spaced. Reprinted from Gallistel and Gelman (2000)

Turning to production studies, a relevant experiment was conducted by Platt and Johnson (1971) and later discussed by Gallistel and Gelman (2000). In the original study, two rats were rewarded for pressing a counter lever at least a target number of times before pressing a second lever. If the press on the second lever occurred too soon, the lever press count was reset to zero.^3^ Figure 3 from their paper (reprinted here as Fig. 4a) shows the proportion of trials for each target number in which the animal made each possible number of presses on the counter lever before pressing the second lever. Figure 4b shows the results from the second rat as presented by Gallistel and Gelman (2000), along with their estimated means and standard deviations (middle panel) and CVs (bottom panel). As can be seen, the figure portrays the CV as approximately constant across all values of the target number of required lever presses.

#### Modeling the behavioral data

We estimated the relative frequency of trials on which each animal made exactly *n*_*r*_ responses in each target numerosity *n*_*t*_ condition of the Platt and Johnson (1971) study using computer graphics software.^4^ We converted the relative frequencies to absolute frequencies by multiplying the relative frequencies by 400, the number of trials at each value of *n*_*t*_ used in the Platt and Johnson study, and considered the sum of these frequencies to be the total number *m* of trials for each target numerosity *n*_*t.*_^5^ For reanalysis of the data, the models we considered are specified by the same equations defined above for the underlying mean and standard deviation of the human data, allowing us to describe both mean estimates and the corresponding variability either as scalar or power-law functions. In this case, we did not consider the two-regimens models, because it is not immediately clear what would correspond to a “density” variable for the Platt and Johnson experimental setting, in which the animal is freely producing responses rather than experiencing an external input that has both density and numerosity characteristics. We adopted a maximum-likelihood estimation approach for estimating the optimal parameters, maximizing the sum of the probabilities of the *n*_*r*_ values over the set of *m* trials for each target numerosity *n*_*t*_ (4, 8, 16, 24). The negative log-likelihood for each target numerosity was defined as:

4 $$ NLL\left(\left\{{n}_r\right\}, mu, sd\right)=\frac{m}{2}\log \left(2\pi {sd}^2\right)+\left(\frac{\sum \limits_i^m{\left({n}_r(i)- mu\right)}^2}{2{sd}^2}\right), $$

where {*n*_*r*_} is the set of numbers of responses made on the *m* trials for the target numerosity, *mu* is the model-derived estimate of the mean of the animal’s numerosity distribution for the given value of *n*_*r*_, and *sd* is the model-derived estimate of the standard deviation of the numerosity distribution. Along with LL values, we also report values for the Akaike information criterion (AIC) and the Bayesian information criterion (BIC), defined as:

5 $$ \mathrm{AIC}=2\ast k\hbox{--} 2\ast LL, $$

6 $$ \mathrm{BIC}=\log (m)\ast k\hbox{--} 2\ast LL, $$

where *k* is the number of parameters used to model the standard deviation of the responses and *m* is the number of data points being estimated. Because the BIC imposes the greater penalty for added parameters, we concentrate on the BIC values in comparing the goodness of fit. To better evaluate the relative performance of the scalar variability models compared with the power variability models, we also quantified the relative likelihood by computing e^(BICscalar – BICpower)/2^, as also discussed in Noorbaloochi, Sharon, and McClelland (2015).

#### Results and discussion

**Fig. 5:**
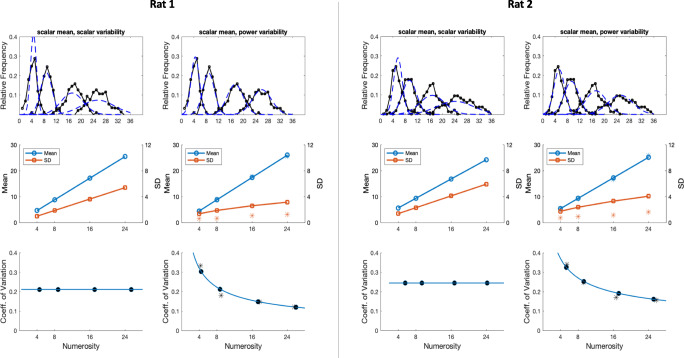
Predictions for models featuring linear mean, with either scalar or power variability. Top panels: response frequency for both rats reported in Platt and Johnson (1971), along with fitted curves resulting from maximum-likelihood estimation. Middle panels: maximum-likelihood estimates of means and standard deviations. Bottom panels: resulting coefficients of variation (CV). Note the decreasing CV trend for the model featuring a power law scaling of estimates variability. Data points marked with asterisks correspond to the empirical means, standard deviations, and CV values

As reported in Table 3, the models with power variability provide a much better account for the data, which is also evident from the fitted curves superimposed over the original data points in the top panels of Fig. 5 (note that plots only show the models featuring linear mean, since the discrepancy with power mean models was minimal). Support in favor of the power variability models is further provided by the extremely large values of the relative likelihoods (for the first rat: 8.30 × 10^82^ in the case of linear mean and 2.72 × 10^85^ in the case of power mean; for the second rat: 2.33 × 10^37^ in the case of linear mean and 4.68 × 10^38^ in the case of power mean). Interestingly, this pattern is exactly what one would expect if behavior in these tasks is based on summation of independent and identically distributed noisy increments. We discuss this finding in more detail below.

**Table 3:** Parameters and goodness of fit statistics for the models describing the data from Platt and Johnson (1971)

Model type	Subj	*s*_*mu*_	*p*_*mu*_	*off*	*s*_*sd*_	*p*_*sd*_	LL	AIC	BIC	*k*
Linear mean, scalar variability	Rat 1	1.04	1	0.42	0.21	1	−3452	6,911	6,927	3
	Rat 2	0.93	1	1.91	0.25	1	−3511	7,028	7,043	
Linear mean, power variability	Rat 1	1.08	1	0.12	0.66	0.48	−3258	6,524	6,545	4
	Rat 2	0.99	1	1.38	0.70	0.55	−3421	6,851	6,872	
Power mean, scalar variability	Rat 1	1.40	0.91	−0.42	0.21	1	−3449	6,906	6,928	4
	Rat 2	0.59	1.14	2.86	0.24	1	−3507	7,021	7,043	
Power mean, power variability	Rat 1	1.68	0.87	−1.32	0.66	0.47	−3249	6,508	6,534	5
	Rat 2	0.56	1.17	2.66	0.70	0.54	−3414	6,838	6,864	

*Note*. The last column reports the number *k* of free parameters for each model

**Table 4:** Empirical data digitally measured from Fig. 3 in Revkin et al. (2008)

*N*	10	20	30	40	50	60	70	80
Mean response	10.05	23.01	32.90	43.01	51.97	60.26	67.10	71.61
Mean CV	0.18	0.27	0.28	0.26	0.24	0.21	0.16	0.11
CV *SE*	0.009	0.018	0.018	0.018	0.011	0.010	0.018	0.009

## General discussion

Our reanalysis of published data from numerosity estimation studies indicates that estimates of numerosity are distributed around the mean, and that variability tends to increase with numerosity, but not always in strict accordance with the idea that variability is a constant function of the mean estimate. Rather, in two studies previously thought to support a constant coefficient of variation we find that the CV tends to decrease with numerosity. We have also reconsidered the data from the discrimination study of Newman (1974), whose own statistical tests also observed a decreasing CV as a function of numerosity. This evidence demonstrates that adherence to Weber’s law is not an invariant property of representations underlying either perception or production of approximate numbers. In further support of this conclusion, it is worth mentioning another study that is inconsistent with the principle of a constant CV in numerosity perception. In this study (Burgess & Barlow, 1983), individual data from two participants performing numerosity estimation with numerosities spanning the range from 10 to 400 were well fit by the model we have called the “simple power variability model,” with estimates of the exponent equal to .75 ± .09 for one participant and .71 ± .05 for the other.

We stress that variability in estimates of numerosity may sometimes exhibit consistency with Weber’s law—it is not our claim that they never to. As one example, the data from Pomè et al. (2019) appears largely consistent with a constant coefficient of variation over the range *n =* 8 to ~80. The study by Whalen et al. (1999) provides an example from a human production study. Thus, it appears that the parametric form of the relationship between numerosity and variability in estimates of numerosity is something that can vary from experiment to experiment.

These observations are consistent with the finding that numerosity judgments are often affected by various perceptual factors other than numerosity (Clayton, Gilmore, & Inglis, 2015; Gebuis, Cohen Kadosh, & Gevers, 2016; Gebuis & Reynvoet, 2012), thus calling into question the existence of a “pure” system, specifically evolved to represent numerosity. Interestingly, in our analysis of the data presented in Revkin et al. (2008) and Newman (1974), the best fitting models indeed assume that estimations of the larger numerosities might have been carried out by relying on density rather than numerosity, a variable whose response variability decreases as a square root of numerosity (Anobile et al., 2014; Pomè et al., 2019). Our simulations cannot rule out the possibility that a pure numerosity system exhibiting the scalar variability pattern is relied on for smaller numerosities, as advocated by Anobile et al. (2014). However, two considerations make us pessimistic about the prospects for this simple two-process account. First, our estimates of the likelihood that the observed data from either Revkin et al. (2008) or Newman (1974) are consistent with these models may be overgenerous, as they fail to take into account within-subject individual differences as a source of variability. Without the full data set for the individual participants, our models cannot capture this source of variability, which could substantially reduce uncertainty about the trend in the CV data. Second, the findings of Burgess and Barlow (1983), where estimates spanning a wide range of numerosities could all be characterized with a simple power variability model, do not seem easy to reconcile with the scalar variability plus sparse versus dense regimen account.

It should also be noted that explicit calibration or feedback after each trial is commonly used in empirical studies (Halberda & Feigenson, 2008; Izard & Dehaene, 2008; Piazza, Izard, Pinel, Le Bihan, & Dehaene, 2004; Revkin et al., 2008). Similarly, reinforcement signals given in animal studies (Platt & Johnson, 1971) provide a fundamental cue that is used for optimal calibration of the responses. Such feedback signals might significantly alter the distribution of responses that would be observed in more ecological settings, thus raising further uncertainty about the natural existence of behavioral patterns strictly following Weber’s law. One can, in fact, imagine that numerosity information is encoded in the brain using various representations, which are flexibly deployed depending on the context and task demands (Siegler & Opfer, 2003) and tuned by signals that support optimization of the use of these representations.

We observed a particularly striking deviation from the scalar variability pattern in the data from the study of Platt and Johnson (1971). There, the data from two rats seemed more consistent with a square-root law variability pattern – a pattern we would expect if each response contributed an independent noisy estimate to a summed estimate of variability. Future research should consider what conditions might promote such a pattern of responding, as opposed to one in which variability in estimates increases closer to linearly with the mean.

In conclusion, we believe that a deeper understanding of numerosity perception will require considering alternatives to the search for evidence of adherence to idealized, essential characteristics: We should also strive to define what could be the underlying mechanisms giving rise to the complex behavioral patterns observed in these studies. Promising results in this direction have been recently achieved by connectionist modeling—for example, by showing how approximate adherence to Weber’s law can emerge in generic neural networks that learn the statistics of their visual environment (Stoianov & Zorzi, 2012; Zorzi & Testolin, 2018), or how developmental trajectories of numerical acuity in children can be simulated by progressive deep learning (Testolin, Zou, & McClelland, 2020). Further research is required to explore these issues more fully, keeping in mind that we must be prudent when characterizing the actual patterns observed in the empirical data. In the context of numerosity estimation, idealizations such as scalar variability and ratio dependence should be conceived as potentially useful descriptive abstractions, without necessarily reflecting an essential characteristic of numerosity judgments.

## Figures and Tables

**Fig. 6:**
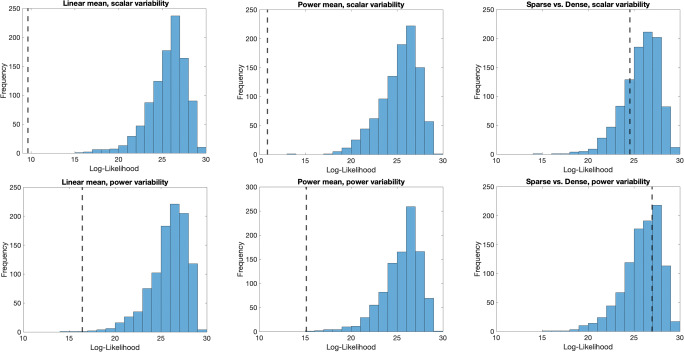
Histograms of the log likelihood of the 1,000 simulated data sets for each model. The vertical dashed line represents the log-likelihood value for the empirical data under each model

## Data Availability

The data we measured from the figures in Revkin et al. (2008) is reported in Table 4. The data we measured from the figures in Platt and Johnson (1971) is made freely available to download at the Open Science Framework (https://osf.io/qb3sm).

## Appendix

### Summed squared errors (SSE) optimization

The MATLAB function *fmincon* was used to minimize the following SSE objective:

7 $$ SSE\left( mu,{mu}_{pred}, CV,{CV}_{pred},n\right)=\sum \limits_n{\left({mu}_{pred}(n)- mu(n)\right)}^2+w\sum \limits_n{\left({CV}_{pred}(n)- CV(n)\right)}^2, $$

where *mu* and *CV* represent the empirical data measured from Figure 1, while *mu*_*pred*_ and *CV*_*pred*_ represent the corresponding values predicted by the model. A weighting factor *w* = 10,000 was included to balance the relative contribution of the mean and CV terms, since these quantities are expressed in a different scale.

### Log-likelihood estimation

In order to consider the actual variability in the estimates of the empirical CV (provided by the error bars in Fig. 1) when judging the goodness of fit, in the case of CV data we also calculated the models’ log likelihood, which was formulated as:

8 $$ LL\left( CV,{CV}_{pred},n,s\right)=\frac{1}{2}\log \left(2\pi {s}^2\right)+\left(\frac{{\left({CV}_{pred}(n)- CV(n)\right)}^2}{2{s}^2}\right), $$

where *s* represents the scaling factor.

The LL values reported in Table 1 have been calculated by considering as scaling factor the standard errors measured from Fig. 1. However, this LL estimate might not be fully justified from a theoretical point of view, so we also implemented an alternative method to assess whether the deviation between the empirical data and the model is greater than we would expect by chance (also see Gao, Tortell, & McClelland, 2011). The underlying idea is to sample synthetic data from each model, and compare the LL of the synthetic data to the LL of the empirical data. To this aim, we generated 1,000 simulated data sets, each one containing simulated responses of 16 “pseudoparticipants” for 20 trials of each numerosity (note that the number of participants and the number of trials were chosen to match those in Revkin et al., 2008). Responses were generated according to the *mu* and *sd* parameters defining each model. For each pseudoparticipant, we then estimated the CV by fitting the cumulative response distribution for each numerosity with the cumulative of a Gaussian distribution function, as in Revkin et al. (2008). We then averaged the estimates across participants to get a mean estimate of the CV along with an estimate of the standard error of the estimates. This allowed to estimate the log likelihood of each simulated data set using the LL equation reported above, using as scaling factor the mean standard error of the simulated CV values. We finally generated LL histograms for each model (shown in Fig. 6), and compared the LL distribution with the LL value from the empirical data (vertical lines in Fig. 6). The index *p* reported in Table 1 measures the proportion of simulations producing larger LL values than the value obtained from the fit to the experimental data.

## Footnotes

1 We reached two authors of the study (M. Piazza and V. Izard) to obtain the relevant data of the target paper. Unfortunately, neither author was able to retrieve the original data. Our digitally measured data is reported in the Appendix for reproducibility.

2 For the lowest numerosity, the bin ranged over the interval [0, 15], while for the higher numerosity the bin ranged over the interval [75, 1000].

3 In addition to resetting the count, the second (but not the first) rat experienced a time-out, further discouraging undercounts.

4 We contacted J. Platt to obtain the relevant data of their original paper, but he reported that the data were no longer available. We also contacted C. R. Gallistel, who kindly provided the data for the second animal reprinted in Gallistel and Gelman (2000). Our estimates for the standard deviation (*SD*) values obtained both from our measurements from Fig. 3 in Platt and Johnson (1971) or from the data points Gallistel provided were quite different from those reported in Gallistel and Gelman (e.g., the *SD* computed for *n* = 24 is around 5, while they report a value of about 10). We discussed this issue with Gallistel, but the discussion did not converge on an understanding of the reason for the discrepancy.

5 The total number of responses *m* generated for each numerosity were, respectively, 396, 368, 358, 382 for the first rat and 379, 397, 353, 301 for the second rat: Shortfall in trials is likely due to trials in which the animals failed to perform the trial at all.

## References

[CR1] Anobile G, Cicchini GM, Burr DC (2014). Separate mechanisms for perception of numerosity and density. Psychological Science.

[CR2] Burgess A, Barlow H (1983). The precision of numerosity discrimination in arrays of random dots. Vision Research.

[CR3] Burr DC, Ross J (2008). A visual sense of number. Current Biology.

[CR4] Butterworth B (1999). *The mathematical brain*.

[CR5] Cantlon JF, Brannon EM (2007). How much does number matter to a monkey (*Macaca mulatta*)?. Journal of Experimental Psychology: Animal Behavior Processes.

[CR6] Clayton S, Gilmore C, Inglis M (2015). Dot comparison stimuli are not all alike: The effect of different visual controls on ANS measurement. Acta Psychologica.

[CR7] Dehaene S (2003). The neural basis of the Weber–Fechner law: A logarithmic mental number line. Trends in Cognitive Sciences.

[CR8] Dehaene S (2011). *The number sense: How the mind creates mathematics*.

[CR9] Ditz, H. M., & Nieder, A. (2016). Numerosity representations in crows obey the Weber–Fechner law. *Proceedings of the Royal Society B: Biological Sciences*, *283*(1827). 10.1098/rspb.2016.0083 10.1098/rspb.2016.0083 PMC4822466 27009227

[CR10] Feigenson L, Dehaene S, Spelke ES (2004). Core systems of number. Trends in Cognitive Sciences.

[CR11] Ferrigno S, Cantlon JF (2017). Evolutionary constraints on the emergence of human mathematical concepts. Evolution of Nervous Systems.

[CR12] Gallistel CR, Gelman R (2000). Non-verbal numerical cognition: From reals to integers. Trends in Cognitive Sciences.

[CR13] Gao J, Tortell R, Mcclelland JL (2011). Dynamic integration of reward and stimulus information in perceptual decision-making. PLOS ONE.

[CR14] Gebuis T, Cohen Kadosh R, Gevers W (2016). Sensory-integration system rather than approximate number system underlies numerosity processing : A critical review. Acta Psychologica.

[CR15] Gebuis T, Reynvoet B (2012). The interplay between nonsymbolic number and its continuous visual properties. Journal of Experimental Psychology. General.

[CR16] Halberda, J. (2011). What is a Weber fraction? https://citeseerx.ist.psu.edu/viewdoc/download?doi=10.1.1.672.2472&rep=rep1&type=pdf

[CR17] Halberda J, Feigenson L (2008). Developmental change in the acuity of the “number sense”: The approximate number system in 3-, 4-, 5-, and 6-year-olds and adults. Developmental Psychology.

[CR18] Izard V, Dehaene S (2008). Calibrating the mental number line. Cognition.

[CR19] Merten K, Nieder A (2009). Compressed scaling of abstract numerosity representations in adult humans and monkeys. Journal of Cognitive Neuroscience.

[CR20] Newman CV (1974). Detection of differences between visual textures with varying number of dots. Bulletin of the Psychonomic Society.

[CR21] Nieder A (2005). Counting on neurons: The neurobiology of numerical competence. Nature Reviews Neuroscience.

[CR22] Noorbaloochi S, Sharon D, McClelland JL (2015). Payoff information biases a fast guess process in perceptual decision making under deadline pressure: Evidence from behavior, evoked potentials, and quantitative model comparison. Journal of Neuroscience.

[CR23] Piazza M, Izard V, Pinel P, Le Bihan D, Dehaene S (2004). Tuning curves for approximate numerosity in the human intraparietal sulcus. Neuron.

[CR24] Platt J, Johnson D (1971). Localization of position within a homogeneous behavior chain: Effects of error contingencies. Learning and Motivation.

[CR25] Pomè A, Anobile G, Cicchini GM, Burr DC (2019). Different reaction-times for subitizing, estimation, and texture. Journal of Vision.

[CR26] Price GR, Palmer D, Battista C, Ansari D (2012). Nonsymbolic numerical magnitude comparison: Reliability and validity of different task variants and outcome measures, and their relationship to arithmetic achievement in adults. Acta Psychologica.

[CR27] Revkin SK, Piazza M, Izard V, Cohen L, Dehaene S (2008). Does subitizing reflect numerical estimation?. Psychological Science.

[CR28] Sella, F., Berteletti, I., Lucangeli, D., & Zorzi, M. (2015). Spontaneous non-verbal counting in toddlers. *Developmental Science*, 1–9. 10.1111/desc.12299 10.1111/desc.12299 25754974

[CR29] Siegler RS, Opfer JE (2003). The development of numerical estimation: Evidence for multiple representations of numerical quantity. Psychological Science.

[CR30] Stoianov I, Zorzi M (2012). Emergence of a “visual number sense” in hierarchical generative models. Nature Neuroscience.

[CR31] Testolin, A., Zou, W. Y., & McClelland, J. L. (2020). Numerosity discrimination in deep neural networks: Initial competence, developmental refinement and experience statistics. *Developmental Science*. 10.1111/desc.12940 31977137

[CR32] Whalen J, Gallistel CR, Gelman R (1999). Nonverbal counting in humans: The psychophysics of number representation. Psychological Science.

[CR33] Wynn K, Cummins D, Allen C (1998). An evolved capacity for number. *The evolution of mind*.

[CR34] Zorzi, M., & Testolin, A. (2018). An emergentist perspective on the origin of number sense. *Philosophical Transactions of the Royal Society B: Biological Sciences*, *373*(1740). 10.1098/rstb.2017.0043 10.1098/rstb.2017.0043 PMC5784047 29292348

